# Supplementary material for: Carbon Quantum Dots-Functionalized UiO-66-NH2 Enabling Efficient Infrared Light Conversion of 5-Hydroxymethylfurfuryl with Waste Ethanol into 5-Ethoxymethylfurfural
Source: Int J Environ Res Public Health. 2022 Aug 22;19(16):10437. doi: 10.3390/ijerph191610437 (PMC9408137; doi:10.3390/ijerph191610437)
Supplement: Supplementary file 1 [file ijerph-19-10437-s001.zip › ijerph-1857470-SI.pdf]

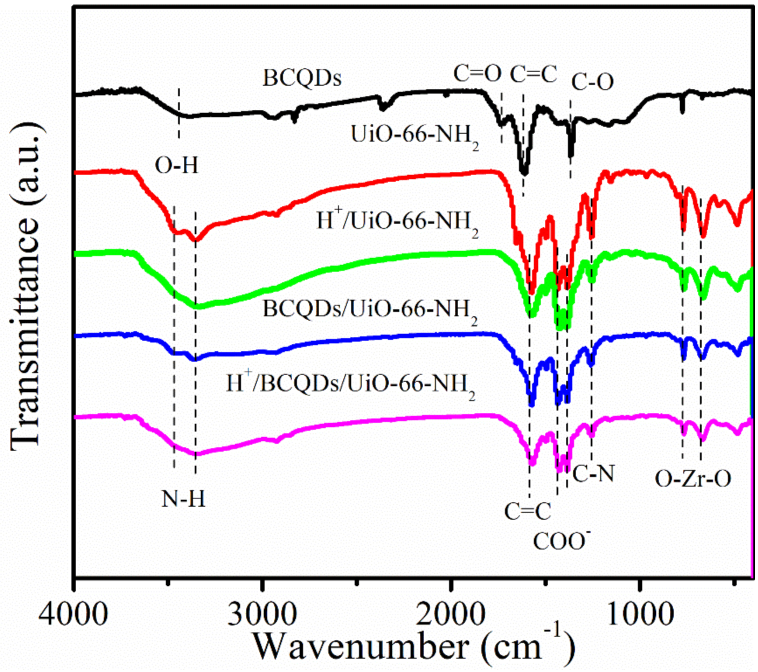

Figure S1. FT-IR spectra of BCQDs, UiO-66-NH_2, H^+/UiO-66-NH_2, BCQDs/UiO-66-NH_2 and H^+/BCQDs/UiO-66-NH_2
